# Deep Eutectic Solvent-Based Emulsion Containing *Piper betle* L. Extract and Hydroxychavicol Prevent Biofilm Development and Surface Adhesion of Avian Pathogenic *Escherichia coli* on Stored Chicken Meat

**DOI:** 10.3390/antibiotics15040328

**Published:** 2026-03-24

**Authors:** Kunchaphorn Ratchasong, Phirabhat Saengsawang, Gorawit Yusakul, Krittika Kabploy, Hemanth Kumar Lakhanapuram, Aliakbur Harudeen, Phitchayapak Wintachai, Thotsapol Thomrongsuwannakij, Ozioma Forstinus Nwabor, Watcharapong Mitsuwan

**Affiliations:** 1 Program in Agriculture and Food Science, College of Graduate Studies, Walailak University, Nakhon Si Thammarat 80160, Thailand; kunchaphorn.ra@mail.wu.ac.th (K.R.); hemanthkumar.la@st.wu.ac.th (H.K.L.); 2 Department of Microbiology and Immunology, Faculty of Veterinary Medicine, Kasetsart University, Bangkok 10900, Thailand; fvetphsa@ku.ac.th; 3 Department of Pharmaceutical Chemistry and Pharmacognosy, Faculty of Pharmaceutical Sciences, Naresuan University, Phitsanulok 65000, Thailand; gorawity@nu.ac.th; 4 Research and Innovation Cluster for Natural Health Products, Naresuan University, Phitsanulok 65000, Thailand; 5 School of Agricultural Technology and Food Industry, Walailak University, Nakhon Si Thammarat 80160, Thailand; krittika.ka@wu.ac.th; 6 Center of Excellence in Innovation of Essential Oil and Bioactive Compounds, Walailak University, Nakhon Si Thammarat 80160, Thailand; 7 Akkhraratchakumari Veterinary College, Walailak University, Nakhon Si Thammarat 80160, Thailand; aliakbur.d@gmail.com (A.H.); thotsapol.th@wu.ac.th (T.T.); 8 School of Science, Walailak University, Thasala, Nakhon Si Thammarat 80160, Thailand; phitchayapak.wi@wu.ac.th; 9 One Health Research Center, Walailak University, Nakhon Si Thammarat 80160, Thailand; 10 Department of Public Health, Maxwell School of Citizenship and Public Affairs, Syracuse University, Syracuse, NY 13244, USA; ofnwabor@syr.edu

**Keywords:** APEC, *Piper betle*, hydroxychavicol, deep eutectic solvent, biofilm, adhesion

## Abstract

**Background:** Avian pathogenic *Escherichia coli* (APEC) contributes substantially to colibacillosis outbreaks in chickens. Because APEC cells readily attach to surfaces and develop biofilms, they pose a notable hazard to poultry production and food safety. This study investigated the antibiofilm and anti-adhesion activities of deep eutectic solvent-based emulsion containing *Piper betle* L. extract (DEPE) and hydroxychavicol, a pure compound isolated from *P. betle* leaves against APEC. **Methods:** Antibiofilm and anti-adhesion activities of DEPE and hydroxychavicol against APEC were investigated. Molecular docking and dynamics simulation of DEPE and hydroxychavicol was conducted. In addition, anti-adhesion activity of DEPE on chicken meat during storage was evaluated. **Results:** DEPE and hydroxychavicol significantly inhibited biofilm formation at sub-MIC, with DEPE achieving up to 80% inhibition and hydroxychavicol up to 69%. At 8 × MIC, DEPE and hydroxychavicol diminished the viability of both early and established biofilms. Furthermore, DEPE and hydroxychavicol reduced APEC adhesion on the surface as observed by SEM. In silico analyses demonstrated the stable binding of hydroxychavicol to adhesion-related proteins, particularly EcpA and FimH, suggesting a possible mechanism for its anti-adhesion activity. At day 5, DEPE at 4 × MIC significantly reduced 63% bacterial adhesion to chicken meat surfaces during storage, while maintaining the meat’s color. **Conclusions:** These findings indicate that DEPE and hydroxychavicol are promising candidates for limiting APEC biofilm formation and surface attachment and may serve as alternative antibacterial agents in poultry-related food safety applications.

## 1. Introduction

The public health issue relates to food safety, as Avian pathogenic *Escherichia coli* (APEC) is one of the bacterial contaminants of poultry meat [1]. This pathogen is also a major cause of colibacillosis in poultry [2]. Consequently, APEC infections contribute to substantial economic losses in the poultry sector and increase the risk of disease transmission to humans through contaminated food products. In addition, APEC possesses zoonotic potential, as several strains share virulence determinants and antimicrobial resistance profiles with human extraintestinal pathogenic *E. coli*, raising concerns about interspecies transmission along the food production and supply chain [3]. Nevertheless, the microbiological quality of frozen poultry products remains a serious concern, as previous studies have documented bacterial contamination in retail chicken meat sold in Dhaka, Bangladesh. In particular, the presence of extended-spectrum β-lactamase (ESBL)-producing *E. coli* in chicken meat represents a growing global public-health challenge [4].

APEC strains express multiple virulence-associated traits, such as adhesins and fimbria structures, which facilitate attachment to host tissues and inert surfaces, promote biofilm development, and support long-term survival along the food chain [5]. In spite of routine antimicrobial treatment and disinfection practices, APEC remains endemic in poultry farms due to its robust biofilm-forming ability, multidrug resistance, and virulence factors that enhance its survival across diverse environmental conditions [6]. Biofilm development plays a central role in the long-term survival and environmental persistence of APEC.

Biofilm development is initiated by the attachment of bacterial cells to the surface, followed by the release of extracellular polymers that assemble into a protective matrix. This process occurs in three stages: initial attachment, maturation into a structured biofilm, and eventual cell dispersion. Biofilms form in response to environmental stress and act as a barrier against antimicrobials, host immune responses, and predation. Furthermore, biofilms facilitate genetic exchange between bacterial cells, thereby accelerating the dissemination of antimicrobial resistance and virulence traits within microbial communities [7]. Biofilm development in APEC is controlled by interconnected genetic pathways that regulate surface attachment, matrix synthesis, quorum-sensing signaling, and tolerance to environmental stress. Some of the key genetic elements, including adhesion and motility genes, play crucial roles in biofilm formation, including *fimH*, *csgABC*, *sfaAS*, *papG*, etc. [8]. Targeting these biofilm and adhesion-associated genes enhances antimicrobial efficacy, as many conventional antibiotics are ineffective against biofilm-embedded cells. As resistance among APEC strains to commonly used antibiotics, particularly fluoroquinolones, tetracyclines, and β-lactams continues to rise, there is therefore a pressing demand for novel antimicrobial approaches that can interfere with APEC biofilm development and address the shortcomings of existing therapies [9].

Plant-based antimicrobials, particularly derived from medicinal herbs, have emerged as viable candidates to replace or supplement synthetic antibiotics. *Piper betle* L. is a well-known medicinal plant traditionally used throughout Southeast Asia and has attracted attention for its antimicrobial activity attributed largely to hydroxychavicol, a phenolic compound present in its leaves [10]. Previous studies indicate that hydroxychavicol compromises bacterial membrane integrity, interferes with quorum-sensing signaling, and limits biofilm development in several Gram-negative species [11]. However, the practical use of essential oils and phenolic compounds is frequently restricted by their limited solubility in aqueous systems and oxidative instability. To overcome these limitations, emulsion-based delivery has been developed to improve solubility, increase antimicrobial activity, and allow for uniform surface coverage in food. Hydrophobic deep eutectic solvents-based emulsions are novel lipid-based formulations that enhance the solubility, stability, and bioavailability of hydrophobic compounds like hydroxychavicol from *P. betle*. Favored for their low toxicity, biodegradability, and high drug-loading capacity, they are well-suited for pharmaceutical, food, and veterinary applications. The deep eutectic solvent-based emulsion of *P. betle* extract (DEPE), corresponding to the T-80-4 formulation described in our previous study [12]. The T-80-4 formulation was selected based on its optimal physicochemical characteristics, including the smallest mean droplet size, highest hydroxychavicol content, narrow polydispersity index, and highest electrostatic stability. Highlighting its potential as a plant-based antimicrobial strategy for enhancing alternative microbiological safety.

Understanding the mechanisms of biofilm formation in APEC allows for precise targeting of bacterial survival strategies, leading to the development of next-generation antimicrobials. This approach not only inhibits biofilm growth but also reduces the risk of resistance and contamination in food systems. This study aimed to assess the inhibitory effects of DEPE and hydroxychavicol on biofilm formation and bacterial adhesion in APEC. Additionally, we also investigated the reduction in bacterial adhesion on chicken meat.

## 2. Results

### 2.1. DEPE and Hydroxychavicol Inhibit Biofilm Formation

**Figure 1:**
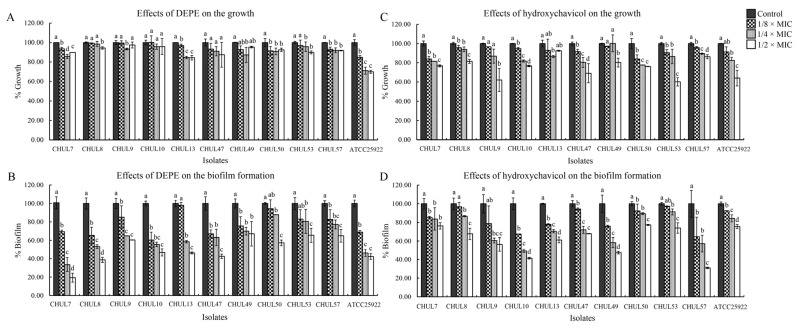
Effects of DEPE (**A**) and hydroxychavicol (**C**) at different concentrations on AEPC growth, biofilm formation, treated with sub-MICs of nanoemulsion (**B**) and hydroxychavicol (**D**). Values are expressed as mean ± SD. One-way ANOVA followed by Duncan’s multiple range test was analyzed. Different lowercase letters (a, b, c, d) indicate statistically significant differences among treatment groups within each isolate at *p* < 0.05.

DEPE and hydroxychavicol demonstrated effective antimicrobial activity against clinically APEC isolates. The previous report by our research team showed the MIC and MBC values of DEPE ranging from 0.06–0.25% *v*/*v*, while the MIC and MBC of hydroxychavicol were 0.25–1.0 mg/mL [12]. Droplet size (including raw DLS data), polydispersity index (PDI), and zeta potential of DEPE, are provided in the Supplementary Data (Table S1 and Figures S1 and S2). This study was further carried out to investigate the activity of DEPE and hydroxychavicol against APEC biofilm formation using a crystal violet assay. The result showed that DEPE and hydroxychavicol at sub-MIC demonstrated significant biofilm inhibition against the clinical isolates (*p* < 0.05) (Figure 1). At 1/2 MIC, DEPE and hydroxychavicol showed approximately 80% (Figure 1B) and 69% (Figure 1D) biofilm inhibition against clinically APEC isolates, respectively. However, DEPE and hydroxychavicol at sub-MICs slightly inhibited the growth of some clinical isolates (Figure 1A,C).

### 2.2. Effect of DEPE and Hydroxychavicol on Bacterial Adhesion

**Figure 2:**
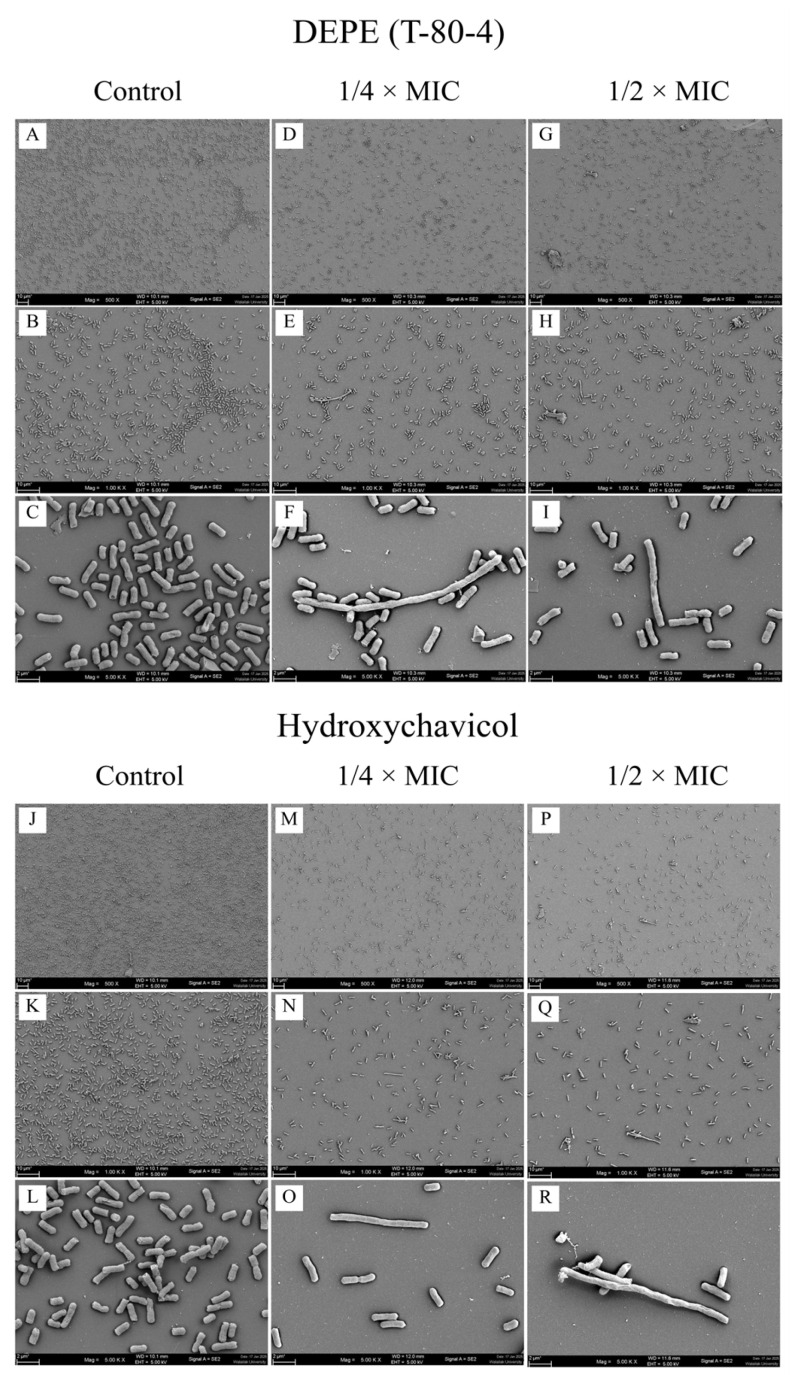
Activity of DEPE (**A**–**I**) and hydroxychavicol (**J**–**R**) on the adhesion of APEC CHUL50 isolate as observed by SEM. Magnifications were revealed as: (**A**,**D**,**G**,**J**,**M**,**P**) = 500×; (**B**,**E**,**H**,**K**,**N**,**Q**) = 1000×; (**C**,**F**,**I**,**L**,**O**,**R**) = 5000×.

The effects of DEPE and hydroxychavicol on the adhesion of APEC cells were observed under SEM. CHUL50 was selected as a representative bacterium. As shown in Figure 2A–C,J–L, bacterial cells in the control group exhibited dense adhesive cells with a normal rod shape. It was observed that APEC cells treated with DEPE (Figure 2D–F,M–O) and hydroxychavicol (Figure 2G–I,P–R) showed a low density of cell adhesion on the surface, compared with the control. Furthermore, the bacteria treated with the compounds, particularly at 1/2 × MIC, displayed clear elongation morphologically (Figure 2I,R), indicating that the treatments disturb the bacterial cell division.

### 2.3. Effects of DEPE and Hydroxychavicol on Established Biofilms

**Figure 3:**
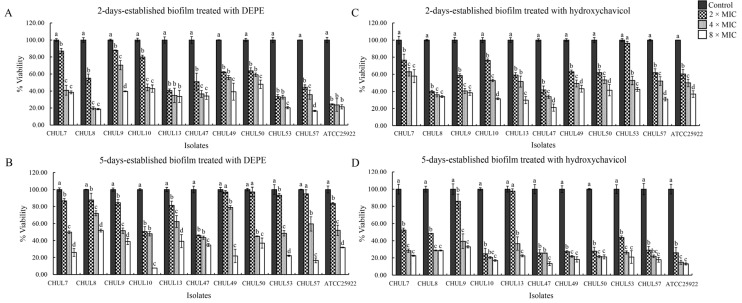
Effects of DEPE on 2-day and 5-day established biofilms (**A**,**B**) and Effects of hydroxychavicol on 2-day and 5-day established biofilms (**C**,**D**). Biofilm viability was assessed after treatment with 2 ×, 4 ×, and 8 × MICs. Values are expressed as mean ± SD. One-way ANOVA followed by Duncan’s multiple range test was analyzed. Different lowercase letters (a, b, c, d) indicate statistically significant differences among treatment concentrations within each isolate at *p* < 0.05.

The activity of DEPE and hydroxychavicol on established biofilms was assessed by MTT assay. As shown in Figure 3A–D, a significant decrease in the viability of young (2-day-old) and mature (5-day-old) biofilms was observed when the established biofilms were treated with both compounds at different MIC values, when compared with the control. For the young biofilm, at the 8 × MIC of DEPE (Figure 3A) and hydroxychavicol (Figure 3C) resulted in a viability reduction to approximately 83% and 79%, respectively. In addition, a reduction in the viability of mature biofilms was detected after treatment with DEPE (Figure 3B) and hydroxychavicol (Figure 3D) at 8 × MIC compared to the control group, with a decrease of approximately 92% and 87%, respectively.

### 2.4. Fluorescence Microscopy of Treated Biofilms

**Figure 4:**
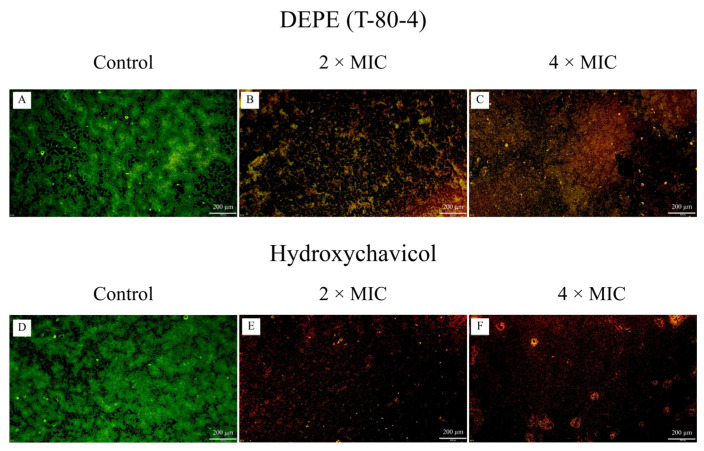
Established biofilm formation of APEC (CHUL50) as observed by fluorescence microscopy visualization of biofilms treated with DEPE (**A**–**C**) and hydroxychavicol (**D**–**F**) at different concentrations. Magnifications were observed at 200×.

Fluorescence microscopy was assessed to confirm the killing activity of DEPE and hydroxychavicol against APEC viable cells in established biofilm. The untreated control biofilms displayed a high density of viable cells, which fluoresced green (Figure 4A,D). In contrast, the established biofilms treated with 2 × MIC and 4 × MIC of the DEPE and hydroxychavicol showed a reduction in cell viability, which was presented in red color (Figure 4B,C,E,F).

### 2.5. In Silico Analysis and Structural Validation of Hydroxychavicol Against APEC Adhesion-Associated Proteins

Structural validation of the four protein models used for molecular docking and molecular dynamics simulation is shown in Table 1. The QMEANDisCo global scores for FimH, CsgA, Yqi, and EcpA were 0.74 ± 0.05, 0.68 ± 0.07, 0.47 ± 0.05, and 0.71 ± 0.06, respectively. Ramachandran plot analysis revealed that FimH exhibited the highest proportion of residues in favoured regions (98.0%), followed by Yqi (95.7%), EcpA (95.3%), and CsgA (93.3%). The percentage of residues in allowed regions ranged from 2.0% to 6.0% across all models. In addition, FimH showed no residues in disallowed regions, while CsgA, Yqi, and EcpA exhibited minimal outlier residues of 0.7%, 0.6%, and 0.5%, respectively.

**Figure 5:**
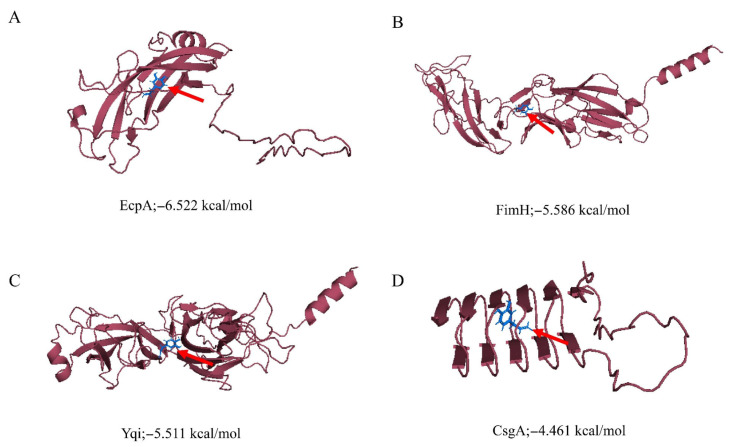
Molecular docking shows the interaction of hydroxychavicol with selected APEC proteins involved in adhesion and biofilm formation: (**A**) EcpA, (**B**) FimH, (**C**) Yqi, and (**D**) CsgA. The red arrows indicate the binding site of hydroxychavicol within the protein structure. Lowest binding affinity values (kcal/mol) are provided below each complex.

**Figure 6:**
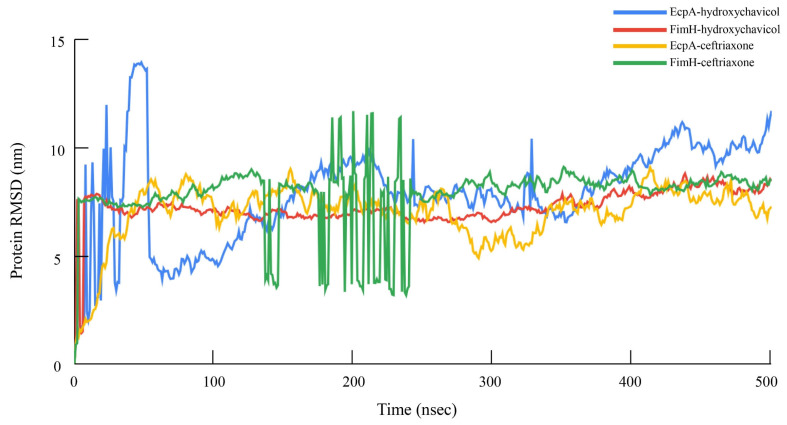
Root mean square deviation (RMSD) analysis of selected APEC proteins involved in adhesion and biofilm formation in the form of complex molecules with hydroxychavicol and ceftriaxone during 500 ns.

**Figure 7:**
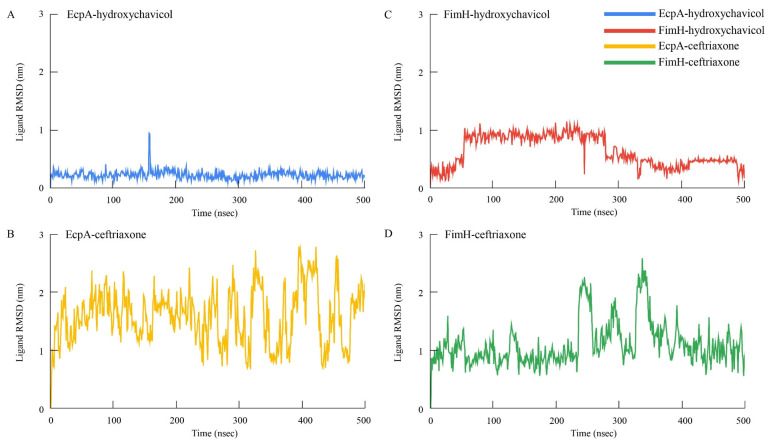
Root mean square deviation (RMSD) plots of hydroxychavicol and ceftriaxone interact with the selected APEC proteins involved in adhesion and biofilm formation during 500 ns. (**A**) RMSD of hydroxychavicol bound to EcpA protein; (**B**) RMSD of ceftriaxone bound to EcpA protein; (**C**) RMSD of hydroxychavicol bound to FimH protein; (**D**) RMSD of ceftriaxone bound to FimH protein.

**Figure 8:**
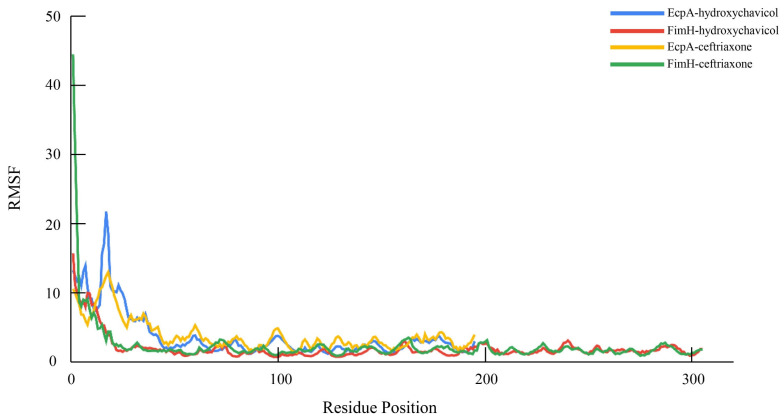
Root mean square fluctuation (RMSF) analysis of EcpA and FimH residues during the molecular dynamics simulation.

**Figure 9:**
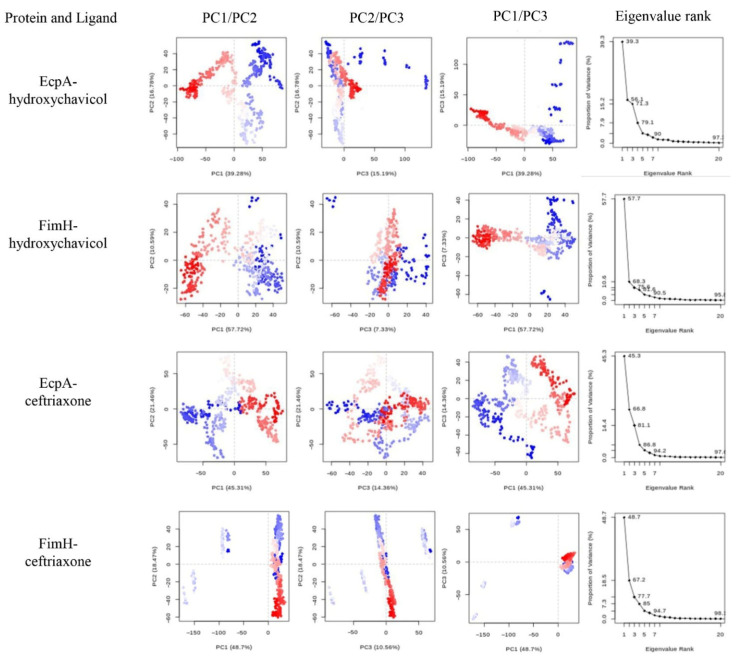
Principal component analysis (PCA) of APEC selected proteins complexed with ligands, and the eigenvalue rank plots display the proportion of variance captured by each principal component.

The binding affinity values of selected proteins were successfully predicted, and the binding site of the hydroxychavicol molecule on each protein structure molecule is presented in Figure 5A–D. The EcpA protein demonstrated the strongest predicted binding affinity, with a binding energy of −6.522 kcal/mol, followed by FimH (−5.586 kcal/mol) and Yqi (−5.511 kcal/mol). CsgA exhibited the weakest predicted interaction, indicated by the least negative score of −4.461 kcal/mol. Ceftriaxone showed the binding energy against EcpA, FimH, Yqi, and CsgA of −6.747, −7.546, −6.989, and −6.513, respectively (Table 2). In addition, structural stability of each protein-ligand complex is presented as RMSD and RMSF. Figure 6 presents the Root Mean Square Deviation (RMSD) values of the selected biofilm-associated proteins during 500 ns. The average RMSD between EcpA and hydroxychavicol was 7.91 ± 2.22 nm, which was nearly similar to average RMSD between EcpA and ceftriaxone (7.02 ± 1.33 nm). In addition, the average RMSD between FimH and hydroxychavicol was 7.25 ± 0.82 nm, similar to the RMSD of ceftriaxone (7.85 ± 1.46 nm). Furthermore, the results showed that after 200 ns, the RMSD values of all proteins revealed greater stability compared to the initial step. The finding indicated that hydroxychavicol had high stability when interacting with the biofilm-associated proteins of APEC. The RMSD of hydroxychavicol (Figure 7) in EcpA had the lowest value (0.07 ± 0.22 nm), followed by FimH (0.64 ± 0.27 nm), suggesting less molecule position change during the simulation time. Conversely, the ceftriaxone complexes showed higher ligand mobility, with RMSD values of 1.58 ± 0.47 nm in EcpA and 1.10 ± 0.38 nm in FimH. These results confirm that hydroxychavicol settled strongly within the binding pockets of the target proteins. Furthermore, RMSF analysis (Figure 8) showed that both EcpA and FimH proteins were highly stable with all residue fluctuations at less than 0.5 nm during the stimulation period. The essential dynamics of the selected proteins were analyzed based on the top three eigenvalues, as shown in Figure 9 (39.9–71.3% for EcpA-hydroxychavicol, 57.7–75.6% for FimH-hydroxychavicol, 45.3–81.1% for EcpA-ceftriaxone, and 48.7–77.7% for FimH-ceftriaxone). Principal component analysis indicates that the structural variance captured by PC3 was minimal compared to the other principal components (Figure 9). FimH-hydroxychavicol showed the lowest variability (7.33%), followed by EcpA-hydroxychavicol (15.19%), FimH-ceftriaxone (10.56%), and EcpA-ceftriaxone (14.36%). It was found that hydroxychavicol may react with the selected proteins by several hydrogen bonds, including 4 bonds with EcpA and 11 bonds with FimH. The positive control showed 8 hydrogen bonds with EcpA and 5 hydrogen bonds with FimH. Of this, in silico results predicted that hydroxychavicol binds most strongly to EcpA, suggesting this protein may be a main target in the antibiofilm mechanism of hydroxychavicol.

### 2.6. Activity of DEPE on APEC Adhesion to Chicken Meat During Storage

**Figure 10:**
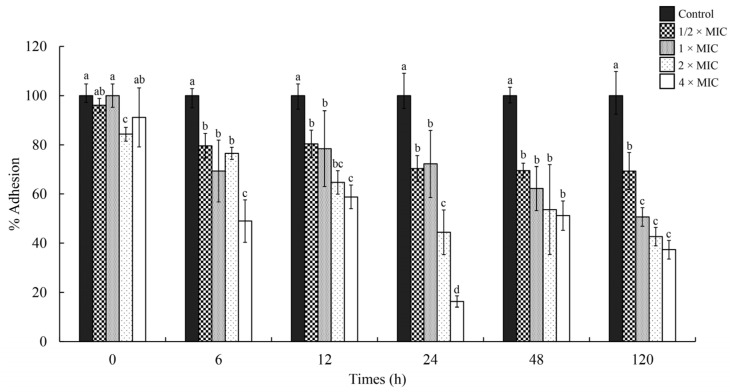
Effects of DEPE on the adhesion of APEC on chicken meat at different concentrations (1/2, 1, 2, and 4 × MIC) during storage at 4 °C. Values are expressed as mean ± SD. One-way ANOVA followed by Duncan’s multiple range test was analyzed. Different lowercase letters (a, b, c, d) indicate statistically significant differences among treatment concentrations within each isolate at *p* < 0.05.

Adhesion is the critical initial step in biofilm formation. Therefore, we evaluated the anti-adhesion activity of DEPE against APEC on chicken meat surfaces. A significant reduction in APEC adhesion on chicken meat was observed at concentrations ranging from 1/2 to 4 × MIC (Figure 10) over 5 days at 4 °C. The result showed that the viability of the APEC decreased within 6 h compared with the control. As observed on days 1, 3, and 5, a decrease in APEC attachment was detected on the surfaces of chicken meat during storage. At the concentration of 4 × MIC, the percentage of bacterial adhesion decreased approximately 83% within 24 h when compared with the control. In addition, the percentage of bacterial adhesion decreased approximately 63% at days 5 during storage at 4 °C.

### 2.7. Evaluation of Chicken Meat Appearance After Treatment with DEPE

**Figure 11:**
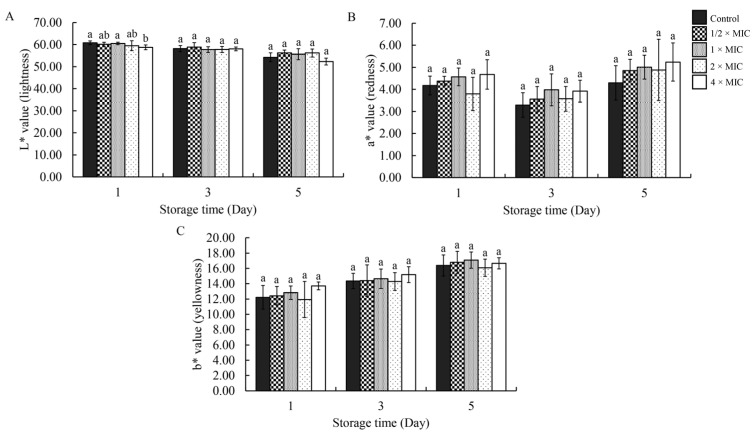
Effect of DEPE treatment on the color stability of chicken meat during storage. The colorimetric parameters (**A**) (L* = lightness), (**B**) (a* = redness), and (**C**) (b* = yellowness) were measured at days 1, 3, and 5 of storage at 4 °C. Values are expressed as mean ± SD. One-way ANOVA followed by Duncan’s multiple range test was analyzed. Different lowercase letters (a, b) indicate statistically significant differences among treatment concentrations within each isolate at *p* < 0.05.

**Figure 12:**
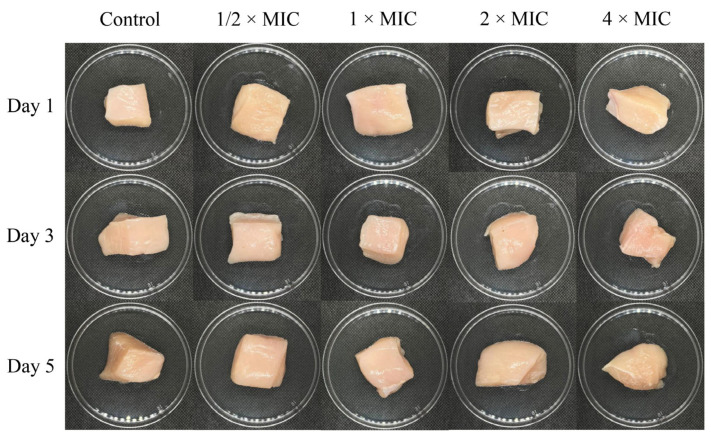
Appearance of chicken meat after treatment with DEPE at different concentrations during storage.

The appearance of chicken meat treated with different concentrations of DEPE during storage is shown in Figure 11 and Figure 12. On day 1 of storage at 4 °C, both the control and all treatment groups exhibited a normal chicken meat color. However, the color of the chicken meat remained unchanged after treatment with DEPE throughout 3 and 5 days of storage. Furthermore, the results showed that the L*, a*, and b* values showed no significant differences when compared with the control group. The color values showed that the DEPE treatment maintained the color quality of the chicken meat.

### 2.8. Effects of DEPE on the Texture Quality of Chicken Meat During Storage

In the present study, DEPE demonstrated high potential in antibiofilm and anti-adhesion activities. In addition, the chicken meat treated with DEPE did not change the color of the chicken meat. Therefore, we assessed the texture quality of chicken meat treated with different concentrations of DEPE. As shown in Table 3, the texture analysis parameters revealed that the hardness, gumminess, and chewiness of chicken meat were significantly affected by both the concentration of the DEPE and the duration of storage. The value of cohesiveness, springiness, and gumminess remained largely unaffected. However, at a DEPE concentration of 4 × MIC, hardness values 1 and 2 were recorded to increase throughout storage compared to the control group. The final parameter analyzed was chewiness, which showed a significant difference in chicken meat treated with 4 × MIC during storage.

## 3. Discussion

It is well-known that APEC is a causative agent of colibacillosis in poultry. The infection is responsible for high morbidity and mortality rates [13]. Controlling APEC infections is a major public health concern, especially considering the current multidrug-resistant (MDR) genes present in APEC strains. Moreover, APEC strains may transfer these resistance genes to pathogenic bacteria such as *E. coli* that cause illness in humans. As a result, a suitable antibiotic management program that addresses both residential and commercial poultry farming is required [14]. The contamination of chicken meat is a significant challenge to food safety and public health. Despite advances in food safety, foodborne illness remains common. Point-prevalence studies of cooked meat products still detect contamination in some samples with major bacterial pathogens, including *Salmonella*, *E. coli* O157:H7, and *Listeria* spp. [15]. Meat is the leading vehicle for foodborne illnesses. The pathogens that cause these infections are typically zoonotic, meaning they can be transmitted from animals to humans [15]. Zoonotic diseases can be introduced at any point along the food chain, from when the animal is raised to the day of slaughter and beyond, up to the moment the meat or poultry product is consumed. Adhesion to surfaces represents the initial step in the pathogenesis and persistence of APEC [16]. Furthermore, this adhesive ability serves as a basis for biofilm development [17]. Biofilm formation serves as a critical virulence mechanism that protects the pathogen from both host immune responses and antibiotic treatment. Consequently, the control of bacterial infections is further complicated by the prevalence of antibiotic resistance [18]. In the present study, we investigated the potential of DEPE against APEC biofilms and the adhesion ability to control the contamination of APEC in chicken during storage.

Biofilm formation is one of the main mechanisms by which APEC survives under stressful conditions and pathogenesis. Therefore, APEC in the form of biofilms often causes chronic infection, making treatment difficult. Recently, we investigated the antibacterial activity of nanoemulsions containing *P. betle*, which have shown activity as vehicles for highly soluble antimicrobials [12]. Importantly, the emulsion base was formed by menthol and lactic acid in a concentrated eutectic solvent (DES) system, a component exhibiting antimicrobial properties and enhanced solubility. This demonstrates that the observed activity of the emulsion may not be solely due to the phytochemicals of *P. betle* but also to the antimicrobial properties of menthol and lactic acid within the DES [12]. Emulsions are delivery systems formed from immiscible oil and water phases stabilized by surfactants, which maintain their stability and viscoelasticity. These systems are extensively used in pharmaceutical technology to encapsulate active ingredients, offering protection and controlled release profiles. Most notably, emulsions are a key strategy for improving the bioavailability of hydrophobic drugs [19,20]. The emulsion base of the observed antimicrobial activity was considered. In a previous study, a formulation control consisting of the nanoemulsion base without *P. betle* extract was evaluated against the same bacterial isolates. MIC and MBC values of the nanoemulsion base were approximately tenfold [12]. This finding indicates that the antimicrobial efficacy observed in the present study is primarily attributable to the bioactive compounds of *P. betle*, rather than to the emulsion carrier itself. A formula containing Tween 80 was selected, which has antibacterial properties [21] that inhibit biofilm formation and reduce bacterial adhesion [22]. Hydroxychavicol, the major phenolic constituent of *P. betle* leaves, is increasingly recognized as a potent antibiofilm agent [23]. It exhibits potent antibacterial activity against Gram-negative and Gram-positive bacteria, including *E. coli* and *Salmonella*. In addition, it has a previously described ability to induce DNA damage [23], and exhibited anti-inflammatory activity [24]. Therefore, the formulation was selected for experiments, including biofilm, food application, and storage studies. Recently, a study reported that the antimicrobial efficacy of menthol-based natural deep eutectic solvents (NADES) incorporating free fatty acids was evaluated against microbial strains. The results revealed pronounced antimicrobial activity against gram-positive bacterial and fungal species. Notably, the menthol and lauric acid formulation (Me: LA, 4:1 molar ratio) demonstrated robust biofilm-disruptive and dispersive activity against methicillin-resistant *Staphylococcus aureus* (MRSA) and *Candida albicans* and also exhibited effective biofilm removal in *E. coli* [25].

Furthermore, the present study demonstrated that DEPE and hydroxychavicol inhibited antibiofilm activity against clinical APEC isolates. These results are consistent with a previous study that reported that the ethanolic extract of *P. betle* significantly inhibited biofilm formation and disrupted mature biofilms of clinical APEC isolates [9]. In addition, hydroxychavicol, the main bioactive component of *P. betle* leaves [26], also showed strong antibacterial and antifungal activities [27]. Hydroxychavicol functions by inducing oxidative stress and DNA damage, a mechanism that leads to bacterial lysis [23] and disrupts cellular processes essential for cell division [12]. It has been accepted that biofilm formation is controlled by a cell-to-cell communication mechanism called quorum sensing (QS) [28]. QS is a cell density-dependent mechanism that regulates key genes involved in biofilm maturation and stability [29]. It has been reported that *P. betle* ethyl acetate extract (PBE) inhibits quorum sensing and biofilm formation in *Vibrio harveyi*. PBE disrupted the initial attachment and architecture of *V. harveyi* biofilms [30]. However, quorum-sensing activity of both compounds was not performed in this study.

The inhibitory effect of DEPE and hydroxychavicol on APEC biofilm adhesion was observed using SEM. The present study confirmed the anti-adhesive properties of both compounds. This reduction in adhesion is a significant finding, as bacterial attachment to surfaces represents the initial and essential step in biofilm initiation and maturation. By preventing this initial step, DEPE and hydroxychavicol effectively reduce biofilm development at its earliest stage. These results corroborate previous reports that ethanolic extracts of *P. betle* significantly reduced the adhesion of APEC on surfaces. The anti-adhesion effects may be attributed to the presence of phenolic compounds, such as hydroxychavicol, which have been reported to affect bacterial cell surface properties [11]. Moreover, a previous study reported that resveratrol, a characterized polyphenolic compound, inhibits biofilm activity. Notably, resveratrol belongs to the same polyphenol group as hydroxychavicol, the bioactive compound investigated in the present study. Resveratrol has demonstrated inhibitory effects on APEC biofilm formation, as confirmed by SEM observations showing a progressive reduction in bacterial adhesion and biofilm density [31].

The ability to eradicate established biofilms is a critical attribute for any antimicrobial agent intended for food safety applications, as bacteria within the EPS matrix are typically highly resistant to antimicrobial agents [32]. In this study, fluorescence microscopy provided compelling evidence that DEPE and hydroxychavicol can effectively penetrate the extracellular polymeric substance (EPS) matrix and exert bactericidal activity against APEC cells. The change in color from green to red in treated biofilms indicates a loss of membrane integrity, as the red dye (propidium iodide) can only enter cells with compromised membranes. Previously, it was similarly reported that the viability of the cells was assessed using confocal laser scanning microscopy (CLSM), where intact cells fluoresced green (SYTO 9) and membrane-compromised cells fluoresced red (propidium iodide) [32]. Moreover, a previous study reported that the activities of *P. betle* leaf extract against biofilm produced by *S. aureus* and *E. coli* [33]. Furthermore, the antibiofilm activity of DEPE, which was shown in the result, may be attributed to the properties of the emulsion system, particularly its enhanced water solubility and droplet size. These characteristics may lead to deeper penetration into the extracellular polymeric substance (EPS) matrix. It has been reported that the nanoemulsion prepared from *Thymus daenensis* oil shows antibiofilm activities against *Acinetobacter baumannii*. The nanoemulsion had strong antibacterial activity against MDR *A. baumannii* isolated strains [34].

The *in silico* docking analysis was conducted to explore the potential affinity of the active compound, hydroxychavicol, for adhesion-associated proteins, in addition to the physical disruption exerted by the emulsion. SEM analysis provides physical evidence, demonstrating a reduction in bacterial cell attachment following treatment with DEPE. Structural validation is an important step. This is because the quality of protein models directly affects the reliability of molecular docking results and the stability of molecular dynamics simulations. Therefore, all four protein models were subjected to stereochemical validation using QMEANDisCo global scores and Ramachandran plot analysis before computational analyses. The QMEANDisCo global scores confirmed that all four protein models were of acceptable stereochemical quality for use in molecular docking and molecular dynamics simulations. In general, a QMEANDisCo score below 0.6 indicates poor model quality, whereas scores above 0.6 suggest acceptable structural quality [35]. In addition, all four protein models had more than 90% residues in the favored regions of the Ramachandran plot, indicating good backbone geometry and supporting the reliability of the structures for analyses [36]. According to molecular docking and dynamics simulation studies, hydroxychavicol shows a high interaction chance with the protein-associated adhesion of APEC, particularly EcpA and FimH. Similarly, a study investigated the binding interactions of the garcinol–FimH complex. The results demonstrated that secondary metabolites of Garcinia species exhibit an inhibitory effect against FimH [37]. In addition, the molecular dynamics simulation indicates that the garcinol–FimH complex is structurally stable throughout the simulation. The RMSD values for both the protein and the complex remained within a narrow range, indicating no conformational changes. Overall, these results support the stability of the garcinol–FimH complex under the simulated conditions [37].

The potential of natural food preservatives. *P. betle* exhibits significant potential as a natural food preservative, demonstrating the capacity to effectively inhibit microbial contamination and extend the shelf life of food products [38]. This study demonstrates that DEPE and hydroxychavicol significantly inhibited APEC biofilm formation and reduced surface adhesion on chicken meat. This is consistent with previous studies that found that ethanolic extracts of *P. betle* significantly inhibited *E. coli* O157:H7 biofilms and prevented bacterial adhesion on beef surfaces stored at 4 °C [39]. In addition, the potential of *P. betle* extracts extends to the sanitation of food processing environments. It was also found that ethanolic extracts of *P. betle* were highly effective in inactivating bacterial biofilms on both pitted and smooth stainless-steel surfaces, exhibiting efficacy comparable to or exceeding that of commercial sanitizers such as sodium hypochlorite and hydrogen peroxide [40]. These demonstrate significant potential as a bio-preservative candidate for controlling APEC contamination in chicken meat. Future studies under industrial processing conditions are necessary to fully validate commercial viability.

The preservation of quality properties of chicken meat after being treated with compounds, particularly color, is a critical determinant of consumer acceptance. In the present study, the application of DEPE at bactericidal concentrations did not induce. Changes in the visual appearance of chicken meat were observed during storage. These findings align with previous research on the effects of two spice extracts and their combination on raw chicken meat throughout storage. Color analysis revealed that treated chicken meat samples maintained stable color parameters during storage, particularly L* values, compared with untreated controls. This preservation of color may be attributed to the antioxidant properties of the bioactive compounds, which help delay oxidative discoloration [41]. These results indicate that the treatment can enhance microbiological safety without negatively affecting meat appearance.

## 4. Materials and Methods

### 4.1. Bacterial Strains and Growth Conditions

Ten APEC isolates obtained from colibacillosis cases in commercial broilers and native chickens were included in the present investigation. Swab samples from the liver, lungs, heart, and spleen of affected chickens were collected from different poultry farms located in southern Thailand. These isolations had been previously characterized by our research group [14]. Ethical approval for all experimental procedures was granted by the Institutional Biosafety Committee of Walailak University, Thailand (Ref. No. WU-IBC-67-029). *Escherichia coli* ATCC 25922 (American Type Culture Collection, Manassas, VA, USA) was employed as the reference strain. For routine propagation, bacterial cultures were grown on tryptic soy agar (Difco, Claix, France) at 37 °C for 24 h and subsequently transferred to tryptic soy broth under identical incubation conditions. Long-term preservation was carried out at −80 °C in broth supplemented with 20% glycerol.

### 4.2. DEPE Preparation and Antimicrobial Agent

The deep eutectic solvent-based emulsion of *P. betle* extract (DEPE) was prepared according to a published protocol [12]. We focus on hydroxychavicol, the main compound of *P. betle*. Regarding extraction yield, the extraction yield of the DEPE system was partially characterized through HPLC-DAD quantification of hydroxychavicol, the primary bioactive compound, which was detected at 4.24 ± 0.01 mg/mL in the T-80-4 formulation [12]. For the T-80-4 formulation, a total volume of 100 mL was made up of 30% hydrophobic deep eutectic solvent (lactic acid and menthol, 1:2), 60% Tween 80 and propylene glycol (1:1), and 10% distilled water. The solution was sonicated at 37 kHz for 10 min before adding 10 g of powdered *P. betle* leaves. Extraction was carried out using microwave irradiation at 1000 W for three pulses of 10 s each, followed by centrifugation at 10,000× *g* for 10 min. The resulting dispersion containing *P. betle* leaves extract exhibited a mean droplet size in the range of 162.60 ± 4.26 nm, which is consistent with reported emulsion systems [42]. The supernatant was collected and stored at 4 °C until analysis. Hydroxychavicol (Tokyo Chemical Industry, Tokyo, Japan) was prepared as a stock solution in dimethyl sulfoxide (100%) and maintained at 20 °C. The particle size, zeta potential, and polydispersity index (PDI) of the T-80-4 formulation (DEPE) were determined using a Zetasizer Nano ZS (Malvern Panalytical, Malvern, UK) as previously described [12]

### 4.3. Activities of Compounds Against APEC Biofilm Formation

Biofilm formation following exposure to DEPE and hydroxychavicol was determined using a crystal violet staining protocol modified from [33]. The minimum inhibitory concentration (MIC) of DEPE against APEC was determined as previously established methods [12]. Bacterial cultures grown overnight in tryptic soy broth with 1% glucose were normalized to 2 × 10^6^ CFU/mL, and 100 µL volumes were inoculated into 96-well microplates. DEPE and/or hydroxychavicol were added at concentrations of 1/2, 1/4, and 1/8 × MIC. Microplates with only the emulsion base served as negative controls. The microplates were incubated at 37 °C for 24 h. Bacterial growth was quantified at an optical density of 600 nm, whereas biofilm biomass was determined at 570 nm following staining. The percentage of bacterial growth and biofilm formation was calculated as follows: (OD of sample treated with compound/OD570 of control) × 100.

### 4.4. Scanning Electron Microscopy (SEM) Analysis of APEC Adhesion After Treatment with DEPE and Hydroxychavicol

Scanning electron microscopy was used to observe the adhesion activities of both compounds against APEC cells. The CHUL50 strain was cultured in tryptic soy broth at 37 °C for 18 h, after which the cell concentration was adjusted to 1 × 10^6^ CFU/mL. Aliquots of the suspension were transferred into centrifuge tubes containing DEPE or hydroxychavicol at 1/2 × MIC and 1/4 × MIC and incubated at 37 °C for 24 h. Cells were subsequently collected by centrifugation at 5000 rpm for 5 min, deposited onto sterile glass coverslips, and air-dried. Samples underwent fixation in 2.5% glutaraldehyde for 2 h, followed by dehydration in ethanol (20–100%) for 30 min. Critical-point-dried specimens were mounted on aluminum stubs and sputter-coated with gold. Bacterial adhesion patterns and surface morphology were visualized using a scanning electron microscope (SEM Zeiss, Munich, Germany).

### 4.5. Eradication of APEC Established Biofilms by DEPE and Hydroxychavicol

The activity of the DEPE and hydroxychavicol in eliminating established APEC biofilms was determined as previously described [43]. Initially, 200 µL bacterial culture was added to a 96-well microtiter plate and incubated at 37 °C for 2 days to establish young biofilms and 5 days to establish mature biofilms. The old medium was removed every 48 h, and fresh medium was added. After incubation, the medium was removed, and the wells were rinsed with PBS. To evaluate the activity of the DEPE and hydroxychavicol, the established biofilms were treated with the emulsion/compound at concentrations ranging from 2 × MIC to 8 × MIC. The emulsion base solution without the compound served as negative control. Then, the medium was substituted with 200-µL PBS containing 10-µL–3-(4,5-dimethylthiazol-2-yl)-2,5-diphenyltetrazolium bromide (MTT) (5 mg/mL; Sigma-Aldrich, Missouri, VA, USA) and further incubated at 37 °C for 2 h. 3-(4,5-Dimethylthiazol-2-yl)-2,5-diphenyltetrazolium bromide is digested by the dehydrogenase enzyme in living bacterial cells within the biofilm, producing insoluble purple formazan. The samples were dissolved in DMSO, and the absorbance was measured at 570 nm. The relative percentage of biofilm formation was calculated as (mean OD570 of treated well/mean OD570 of control well) × 100.

### 4.6. The Activities of Both Compounds in Reducing APEC Viability Within Established Biofilms by Fluorescence Microscopy

Fluorescence microscopy was used to confirm the killing activity of DEPE and hydroxychavicol against the APEC CHUL50 strain embedded in the established biofilm. The samples were stained with 100 μL of a dual fluorescent dye solution containing acridine orange (AO) and propidium iodide (PI). Staining was performed for 10 min at room temperature in the dark. Under fluorescence microscopy, live bacterial cells appeared green, while dead cells appeared red.

### 4.7. Structural Validation and In Silico Analysis

The 3D structures of APEC adhesion-related proteins, namely FimH (UniProt: Q84DW0), CsgA (A0A0H2YXJ4), Yqi (A0A0H2Z2U8), and EcpA (A1A7X6), were obtained from the UniProt database (https://www.uniprot.org). The structural quality of all four protein models was assessed using QMEANDisCo global scores via the SWISS-MODEL server (https://swissmodel.expasy.org/), and Ramachandran plot analysis was performed using the RamPlot web server (https://www.ramplot.in/). The molecular structure of hydroxychavicol (PubChem CID: 70775) was downloaded from PubChem (https://pubchem.ncbi.nlm.nih.gov) and used as the test ligand, while ceftriaxone (PubChem CID: 5479530) served as a reference compound for comparative docking analysis. Receptors and ligands were docked using Autodock Vina (Galaxy version 1.5.7) [44]. Molecular docking was performed in a grid box of 100 × 100 × 100 Å. The best molecular model between hydroxychavicol and each docked protein was selected for molecular dynamics simulation based on the lowest binding energy values. Molecular dynamics simulations were processed using tools on the Galaxy Europe server (https://usegalaxy.eu). Ligand–receptor complexes with low binding energy scores were selected as used structures for molecular dynamics simulations. Briefly, the docked hydroxychavicol-selected protein complexes were prepared in PyMOL (version 3.1.6.1). The prepared complex was processed to generate system topology files and position restraint parameters using the Simple Point Charge (SPC) water model and the Optimized Potentials for Liquid Simulations–All Atom (OPLS/AA) force field. Each protein-ligand complex was configured in a triclinic box (1 nm dimensions) and then solvated under a generic three-point (SPC) model with a neutralized system. In addition, the complex was performed energy minimization with the steepest descent algorithm and Fast smooth Particle-Mesh Ewald (SPME) electrostatics (number of steps: 5 × 10^5^, EM tolerance: 10^4^, and maximum step size: 10^−2^ nm). Following, the obtained potential energy (kJ/mol) during a number of steps of energy minimization was assessed by plotting. Then, the equilibrations under an isothermal-isochoric (NVT) ensemble and an isothermal–isobaric (NPT) ensemble were performed. For the NVT, the leap-frog algorithm for integrating Newton’s equations of motion was set under the condition of 300 K, 0.002 step length (ps), 10^4^ of the number of steps that elapse between saving data points, and 5 × 10^6^ of the number of steps for the simulation. In addition, NPT was also performed under the same conditions as the NVT. Moreover, the main simulation was carried out at 300 K, 0.001 step length in ps, 5 × 10^3^ of numbers of steps that elapse between saving data points, and 5 × 10^6^ of numbers of steps for the simulation. The obtained structures and trajectories of each simulation were used to analyze the root mean square deviation (RMSD) and root mean square fluctuation (RMSF). In addition, the principal component analysis (PCA) and hydrogen bond analyses were performed.

### 4.8. Inhibitory Activity of DEPE on Adhesion of APEC on Chicken Meat

The effect of DEPE on APEC attachment to commercial chicken meat was evaluated using chicken samples obtained from local retail markets. Under sterile conditions, the meat was cut into cubes of approximately 1 cm^3^, rinsed with distilled water, and surface-sterilized by immersion in ethanol for 10 min to minimize background microbial contamination. A suspension of the APEC CHUL50 strain was prepared in phosphate-buffered saline at a final concentration of 2 × 10^6^ CFU/mL. Individual meat portions (25 g) were inoculated with the bacterial suspension and treated with DEPE at concentrations ranging from 1/2 × MIC to 4 × MIC. All treatments were prepared in PBS. Samples were stored at 4 °C and analyzed after 0, 6, 12, 24, 48, and 120 h of incubation. Following storage, adhered and surviving bacteria were quantified by serial tenfold dilution in PBS and plating on Eosin Methylene Blue agar. Colony counts were recorded after incubation at 37 °C.

### 4.9. Effect of DEPE Concentration on the Visual Appearance of Chicken Meat During Storage

Effects of DEPE on chicken meat appearance were carried out using commercially fresh chicken breast that was sliced into 40 mm × 40 mm × 20 mm pieces. The sample was treated with different concentrations of DEPE (0.5, 1, 2, and 4 × MIC). All samples were stored at 4 °C for 1, 3, and 5 days. The sample color was measured using a colorimeter (ColorFlex EZ, HunterLab, Reston, VA, USA). The measurements were recorded in the CIELAB color space, which consists of three parameters: L* (lightness), a* (positive values for red, negative values for green), and b* (positive values for yellow, negative values for blue).

### 4.10. Evaluation of Physical Characteristics of Chicken Meat During Storage

The physical properties of chicken meat treated with DEPE were assessed. Chicken meat was aseptically sliced into pieces measuring approximately 40 mm × 40 mm × 20 mm, as described [45]. Samples were treated with DEPE at concentrations ranging from 1/2 to 4 × MIC and stored at 4 °C for varying durations (1, 3, and 5 days). A texture analyzer (LLOYD Instrument LR5K, Lloyd Instruments Ltd., Bognor Regis, UK) equipped with a 1/4 spherical stainless-steel probe was used to evaluate textural parameters. The test was performed under 60% compression with a crosshead speed of 1 mm/s and a 30-s holding time after probe penetration. The measured parameters included hardness (N), cohesiveness, springiness (mm), gumminess (N), and chewiness (Nmm). Hardness was taken as the maximum force during the first compression. Cohesiveness was calculated as the ratio of the area under the second to the first compression curve (A_2_/A_1_). Springiness was defined as the distance recovered between the end of the first compression and the start of the second compression. Gumminess was calculated as hardness × cohesiveness, and chewiness as hardness × cohesiveness × springiness.

### 4.11. Statistical Analysis

The recorded data from the experiments were analyzed using descriptive statistics, including mean and standard deviation (S.D.). The comparison of continuous variables among experimental groups was analyzed by one-way ANOVA using the Statistical Package for Social Sciences (SPSS) program version 26. The difference significance of the variable from one-way ANOVA was further tested using Duncan’s test. All statistical analyses were performed under a 95% confidence interval, and a *p*-value < 0.05 was considered for statistical significance.

**Limitations and recommendations:** The limitations of this study include that the assessment of meat quality was limited to visual appearance, texture, and color parameters, without a comprehensive sensory evaluation. Future studies should focus on extended storage periods, detailed mechanistic analyses, and consumer acceptance testing to further validate the application of DEPE for improving poultry food safety.

## 5. Conclusions

In the present study, DEPE and hydroxychavicol exhibited strong antibiofilm and anti-adhesion activities against APEC. Both compounds effectively inhibited biofilm formation at sub-MIC and significantly reduced the viability of young and mature biofilms. SEM analyses confirmed a reduction in bacterial adhesion. In addition, molecular docking and molecular dynamics simulations demonstrated stable interactions between hydroxychavicol and protein-associated adhesion, particularly EcpA and FimH. Importantly, DEPE significantly reduced APEC adhesion on chicken meat. The application of DEPE preserved the appearance and maintained the color of chicken meat during storage. Overall, these findings provide preliminary evidence that DEPE and hydroxychavicol may contribute to reducing APEC contamination and warrant further investigation as alternative antimicrobial approaches.

## Figures and Tables

**Table 1:** Structural validation of protein models used for molecular docking and molecular dynamics simulation.

Proteins	Parameters			
	QMEANDisCo Global Score	Ramachandran Favoured	Ramachandran Allowed	Ramachandran Outliers
FimH	0.74 ± 0.05	98.0%	2.0%	0%
CsgA	0.68 ± 0.07	93.3%	6.0%	0.7%
Yqi	0.47 ± 0.05	95.7%	3.7%	0.6%
EcpA	0.71 ± 0.06	95.3%	4.1%	0.5%

**Table 2:** Comparison of binding energies between hydroxychavicol and ceftriaxone during the interaction with selected APEC proteins involved in adhesion and biofilm formation.

Proteins	Binding Energy (ΔG) (kcal/mol)	
	Hydroxychavicol	Ceftriaxone
EcpA	−6.522	−6.747
FimH	−5.586	−7.546
Yqi	−5.511	−6.989
CsgA	−4.461	−6.513

**Table 3:** Effects of DEPE on texture parameters of chicken meat during storage.

Texture Parameters	Concentration	Storage (Days)		
		Day 1	Day 3	Day 5
Hardness1 (N)	Control	21.2950 ± 1.6138 ^a^	9.4168 ± 0.3257 ^a^	14.6289 ± 0.3199 ^a^
	1/2 × MIC	15.3527 ± 3.3958 ^c^	18.4749 ± 1.1580 ^b^	19.5733 ± 0.2657 ^a^
	1 × MIC	19.6379 ± 0.8817 ^ab^	16.7759 ± 5.4373 ^ab^	12.5340 ± 0.0431 ^a^
	2 × MIC	18.3402 ± 0.4058 ^bc^	17.7981 ± 8.5418 ^b^	15.1549 ± 5.7622 ^a^
	4 × MIC	25.5449 ± 4.0174 ^a^	21.1863 ± 0.6852 ^b^	15.7154 ± 5.9618 ^a^
Hardness2 (N)	Control	16.8266 ± 0.1739 ^a^	9.1591 ± 0.2488 ^a^	12.4941 ± 0.3374 ^a^
	1/2 × MIC	12.7340 ± 2.7801 ^b^	16.2984 ± 1.4974 ^a^	16.1294 ± 0.1681 ^a^
	1 × MIC	15.4333 ± 1.8872 ^ab^	14.1309 ± 3.4529 ^a^	10.8222 ± 0.0206 ^a^
	2 × MIC	15.7760 ± 0.4326 ^ab^	15.4937 ± 7.1847 ^a^	12.3303 ± 4.905 ^a^
	4 × MIC	21.3627 ± 3.2848 ^a^	16.3500 ± 0.3159 ^a^	13.2720 ± 4.7290 ^a^
Cohesiveness	Control	0.3223 ± 0.0135 ^a^	0.5236 ± 0.0422 ^a^	0.3340 ± 0.0184 ^a^
	1/2 × MIC	0.3202 ± 0.0156 ^a^	0.4435 ± 0.0278 ^ab^	0.3622 ± 0.0155 ^a^
	1 × MIC	0.3280 ± 0.0453 ^a^	0.4369 ± 0.0836 ^ab^	0.31864 ± 0.0123 ^a^
	2 × MIC	0.3518 ± 0.0502 ^a^	0.4476 ± 0.1017 ^ab^	0.3335 ± 0.0596 ^a^
	4 × MIC	0.3736 ± 0.0123 ^a^	0.3429 ± 0.0116 ^b^	0.3814 ± 0.0450 ^a^
Springiness Index	Control	0.5280 ± 0.0053 ^a^	0.6371 ± 0.0520 ^a^	0.6752 ± 0.1677 ^a^
	1/2 × MIC	0.5480 ± 0.01590 ^a^	0.7403 ± 0.2314 ^a^	0.6799 ± 0.0681 ^a^
	1 × MIC	0.5052 ± 0.0151 ^a^	0.6918 ± 0.2607 ^a^	0.6722 ± 0.0224 ^a^
	2 × MIC	0.5452 ± 0.0544 ^a^	0.5829 ± 0.1045 ^a^	0.6681 ± 0.1138 ^a^
	4 × MIC	0.5339 ± 0.0130 ^a^	0.5431 ± 0.0125 ^a^	0.6774 ± 0.0661 ^a^
Gumminess (N)	Control	6.8415 ± 0.2320 ^a^	4.9439 ± 0.5681 ^a^	4.4390 ± 0.3591 ^a^
	1.2 × MIC	4.9687 ± 1.3271 ^a^	8.2263 ± 1.0272 ^a^	6.5523 ± 0.4648 ^b^
	1 × MIC	6.4819 ± 1.1792 ^a^	7.0500 ± 1.2650 ^a^	3.9944 ± 0.1675 ^a^
	2 × MIC	6.4310 ± 0.7788 ^a^	7.9597 ± 4.0598 ^a^	7.8972 ± 0.3253 ^c^
	4 × MIC	9.5926 ± 1.8161 ^b^	7.2573 ± 0.0112 ^a^	4.5050 ± 1.0991 ^a^
Chewiness (Nmm)	Control	36.1945 ± 1.5659 ^a^	31.8657 ± 6.2211 ^a^	26.5946 ± 0.4695 ^a^
	1/2 × MIC	26.9985 ± 6.5349 ^a^	63.4033 ± 26.7170 ^a^	41.1795 ± 0.8216 ^d^
	1 × MIC	32.9845 ± 6.9557 ^a^	46.6986 ± 8.3147 ^a^	26.1912 ± 0.5238 ^a^
	2 × MIC	35.5540 ± 7.7482 ^a^	45.8385 ± 24.1069 ^a^	21.7687 ± 3.8608 ^b^
	4 × MIC	51.5159 ± 10.960 ^b^	39.4783 ± 0.8509 ^a^	37.0199 ± 1.9057 ^c^

Note: Different lowercase letters (a–d) in each row indicate significant differences among chicken meat treated with different concentrations of DEPE. One-way ANOVA followed by Duncan’s multiple range test was analyzed. A significant difference was considered at *p* < 0.05.

## Data Availability

The original contributions presented in this study are included in the Supplementary Materials. Further inquiries can be directed to the corresponding author.

## Supplementary Materials

The following supporting information can be downloaded at: https://www.mdpi.com/article/10.3390/antibiotics15040328/s1, Figure S1: Zeta potential of DEPE (T-80-4), measured in triplicate by dynamic light scattering at 25 °C. (A) Replicate 1: −45.30 mV, (B) Replicate 2: −45.70 mV, (C) Replicate 3: −47.50 mV; Figure S2: Particle size distribution of DEPE (T-80-4) measured in triplicate (A-C); Table S1: Raw dynamic light scattering (DLS) data of the Deep Eutectic Solvent-Based Emulsion of *Piper betle* L. Extract (DEPE; T-80-4).
