# Supplementary material for: Deep Eutectic Solvent-Based Emulsion Containing Piper betle L. Extract and Hydroxychavicol Prevent Biofilm Development and Surface Adhesion of Avian Pathogenic Escherichia coli on Stored Chicken Meat
Source: Antibiotics (Basel). 2026 Mar 24;15(4):328. doi: 10.3390/antibiotics15040328 (PMC13113980; doi:10.3390/antibiotics15040328)
Supplement: Supplementary file 1 [file antibiotics-15-00328-s001.zip › antibiotics-4182483-supplementary.pdf]

Table S1. Raw dynamic light scattering (DLS) data of the Deep Eutectic Solvent-Based Emulsion of *Piper betle* L. Extract (DEPE; T-80-4)

Measurement	X1	X2	X3	Mean ± SD
Particle size (nm)	162.20	168.00	157.60	162.60 ± 4.26
Polydispersity index (PDI)	0.45	0.47	0.45	0.46 ± 0.001
Zeta potential (mV)	−45.30	−45.70	−47.50	−46.17 ± 1.0

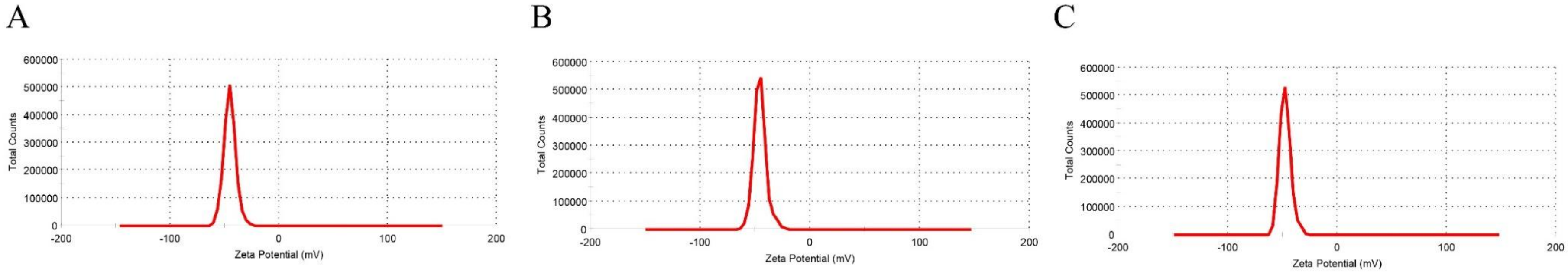

Figure S1. Zeta potential of DEPE (T-80-4) , measured in triplicate by dynamic light scattering at 25°C. (A) Replicate 1: −45.30 mV, (B) Replicate 2: −45.70 mV, (C) Replicate 3: −47.50 mV.

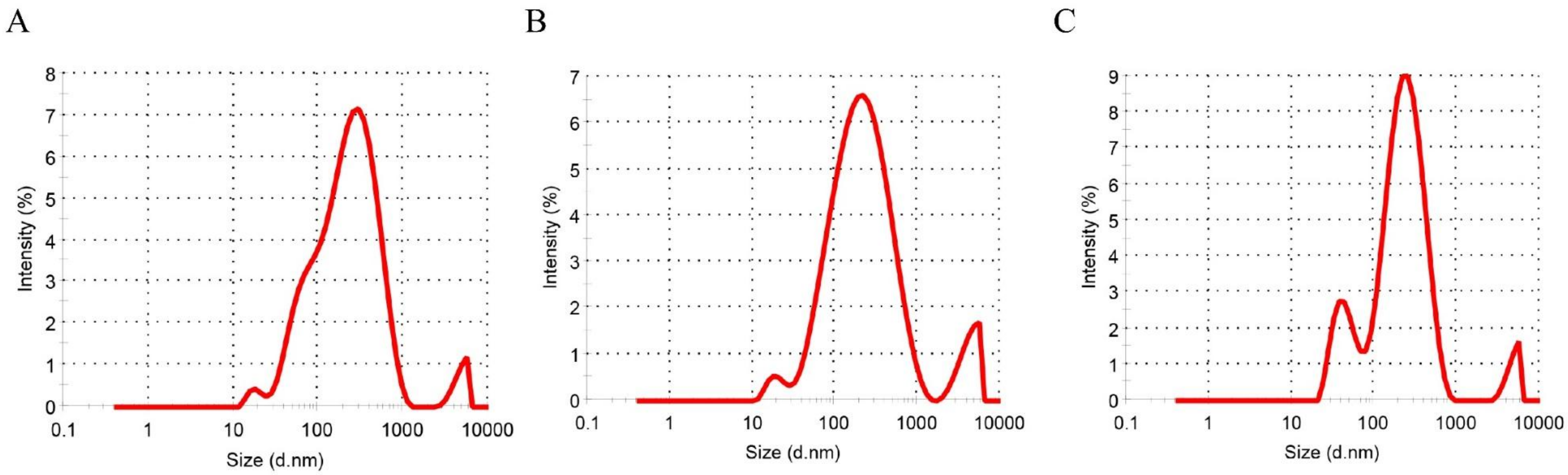

Figure S2. Particle size distribution of DEPE (T-80-4) measured in triplicate (A-C).
